# Indispensable Orthopædies

**Published:** 1915-12

**Authors:** 


### Reviews of Books.

**Indispensable Orthopædics.** By F. Calot. Translated from the Sixth French Edition by A. H. Robinson, M.D., and Louis Nicole. Pp. 1175. London : Baillière, Tindall and Cox. 1914. Price 21s. net.

We think anyone taking up this book may be appalled by its size, and think if all this knowledge is really indispensable then he has not the time to study it. The book embraces the surgery of the bones and the joints, as the American works on orthopædic surgery do, and as Mr. Tubby's new edition of his orthopædic surgery does. This we regret, as we think orthopædic surgery should be the surgery of deformities only.

There is, however, much of considerable interest in this book of M. Calot's, and large though it is, it does well repay careful reading. Many of the ideas are novel to British surgeons, and demand very careful consideration. The author is, of course, well known in connection with the rapid, forcible correction of the curvature of Pott's disease, which he so strongly advocated some years ago, and was tried by many surgeons in this country, but has now, even by Calot, been abandoned.

But his remarks on the gradual correction of the deformity of Pott's disease are most instructive, and his method of treating the disease well worth careful attention. The form of plaster jacket he applies seems to us by far the best with which we are acquainted, but it is only used in addition to a prolonged period of recumbency, and after it.

The author is very much opposed to operation in spinal abscess, and considers the aspiration method is the only treatment which should be undertaken. The aspiration method seems well worth adoption in some cases, but we doubt if it is as good as the free clearing out of a large psoas abscess, as usually practised by British surgeons.

Operation in the paraplegia of Potts' disease is also strongly condemned. We feel sure it is too often undertaken without a trial of prolonged rest and hyperextension of the spine, but we hardly think it would be right to say it is never indicated. M. Calot seems to have been very successful with hyperextension carried out by his special method, and very rapidly so, in some cases, as though the opening out of the curvature had at once relieved pressure on the cord.

The author's treatment of genu valgum and varum does not seem to us a reasonable one. He bends the limb at the knee-joint, so as to correct the deformity. The manipulation may have to be repeated several times, and the limb is fixed in position for many months. If the genu valgum or varum is due, as it always is, to a deformity in the bones (usually the leg bones) surely a forcible bending of the limb at the knee-joint can only stretch the lateral ligament. But of course it is possible that if the leg is fixed in a plaster case for several months, and all pressure removed from it by preventing the child from walking, the bones may grow straight. We should certainly prefer to treat a case bad enough for this manipulation method, by osteotomy, after the period of softening of the bones was over.

In an appendix to the book a very valuable account is given of the new method of treating scoliosis by the American surgeon

Abbott. He corrects the curvature by his method even in quite bad cases, and they have been followed for several years, and have not been found to relapse. The patient is suspended on a hammock with the spine well flexed, and the curvature is corrected by traction with slings, and then the spine fixed in a plaster jacket. A special frame has been constructed by Abbott for the correction of the curvature.

